# Treatment options of Adolescent Gestational Diabetes: Effect on Outcome

**DOI:** 10.12669/pjms.37.4.3966

**Published:** 2021

**Authors:** Shaymaa Kadhim Jasim, Hayder Al-Momen, Maisaa Anees Wahbi

**Affiliations:** 1 Shaymaa Kadhim Jasim Department of Obstetrics and Gynecology, College of Medicine, University of Baghdad, Bab Al-Moaddam, Baghdad, Iraq; 2 Hayder Al-Momen Department of Pediatrics, Al-Kindy College of Medicine, University of Baghdad, Al-Nahda square, Baghdad, Iraq; 3 Maisaa Anees Wahbi Department of Obstetrics and Gynecology, Baghdad Teaching Hospital, Medical City Health Directorate, Ministry of Health, Bab Al-Moaddam, Baghdad, Iraq

**Keywords:** Complications, Gestational diabetes, Insulin, Lifestyle, Metformin

## Abstract

**Objectives:**

Teenage pregnancy with gestational diabetes mellitus (GDM) offers a real challenge to the health system and needs a special care. We aimed to evaluate possible obstetrical and neonatal adverse events of different treatment protocols in adolescent GDM including lifestyle, metformin (MTF), and insulin.

**Methods:**

All teen pregnant women ≤ 19 years old visiting Baghdad Teaching Hospital throughout four years (from June 1, 2016 till May 31, 2020) diagnosed with GDM were included in this cohort study and followed-up closely throughout pregnancy and after delivery. Included adolescents were put on lifestyle alone during the first week of presentation. Adolescents who reached target glucose measurements were categorized into lifestyle group, while other adolescents were randomly allocated into MTF and insulin groups. Also, adolescent pregnant women without GDM were recruited as control group using computer randomization.

**Results:**

The GDM (110 cases) and control (121 individuals) groups had matched general features at recruitment except for diabetes family history. Also, GDM treatment groups had matched features. Glycemic readings (fasting and random) was significantly (p< 0.05) higher in insulin group having odds ratio (OR) of 1.41, and 1.57, respectively. In MTF group, significant protective OR was found in preeclampsia (OR=0.76, p< 0.05). MTF showed non-significant protective OR regarding prematurity and five minutes Apgar score>7 [(OR=0.83, p=0.24), and (OR=0.94, p=0.73), respectively], and significant protective association with large for gestational age and admission to neonatal intensive unit. Insulin had significantly higher prematurity, small for gestational age, and hypoglycemia [OR=1.89, 2.53, and 2.84, respectively].

**Conclusion:**

Metformin (MTF) showed less pregnancy and neonatal complications in adolescent GDM than insulin and lifestyle.

## INTRODUCTION

Teenage (teen) or adolescent period lies within the age range of 10-19 years.^1^ Adolescents have a natural rebellion to medical treatment and doctors` instructions which in turn may lead to more complications with higher frequencies if gestational diabetes mellitus (GDM) is added to the equation of pregnancy.^2^

Pregnancy is a potential risk of glucose intolerance, and insulin sensitivity is further decreased with progression of time till the point of non-matching between the secreted insulin and insulin resistance is reached to declare occurrence of gestational diabetes. On the other hand, teenage pregnancy carries specific hazards affecting pregnancy events and newborn parameters. Accordingly, many health systems have increasing efforts to find out the best approach to deal with such pregnancies.^1^,^2^

Based on the idea of poor compliance in adolescents and supported by the expected adverse obstetric and neonatal events provoked by both gestational diabetes and young (teen) age group, we tried to conduct this prospective cohort to evaluate possible complications occurred during pregnancy, delivery, and early neonatal period in gestational diabetic adolescent women with regard to the main treatment options involving lifestyle, insulin, and metformin (MTF).

## METHODS

Throughout four years starting on June 1, 2016 till May 31, 2020, all adolescent (≤ 19 years) singleton pregnant women with gestational diabetes visiting Department of Obstetrics and Gynecology at Baghdad Teaching Hospital of Medical City Health Directorate in Baghdad, Iraq was included in the study.

In general, the average age of pregnancy in Iraq is 25.7 year`s old.^3^ GDM was diagnosed after 20 weeks` gestation according to the International Association of Diabetes in Pregnancy Study Groups by fasting venous plasma sugar > 91.8 mg/dl, or postprandial glucose either at one or two hours > 180 mg/dl, and > 153 mg/dl, respectively when using oral glucose tolerance test (75 mg).^4^

Enrolled adolescent mothers were requested to measure their glucose level daily during the first week of enrolment and at least twice a week on regular basis afterwards, eight hours after fasting and one hour postprandial using home glucose measuring gadgets of Accu-Chek^®^ Performa from Roche Diabetes Care, Inc.

The hospital guidelines recommended fasting glucose measurements to be (70-90 mg/dl) while random blood sugar one hour postprandial should have been < 140 mg/dl as a target point. Glycated hemoglobin (HbA1c) was not routinely done due to limited resources.

During the first week of presentation, lifestyle management (including proper education, physical activity, and dietary management) was adopted as the only treatment option for the all. Treatment trajectory was decided by the attending obstetrician at the end of the first week of presentation. If the teenage women reached the above-mentioned targets of glucose measurements, they would continue on lifestyle option alone, while the rest of included women were divided into two equal groups using random selection method, one group was treated with insulin (and lifestyle), and the other with MTF (and lifestyle). Depending on pre-pregnancy body mass index (P-BMI) which was calculated retrospectively, diabetic diet estimation was encouraged for normal weight individuals (P-BMI= 18.5-24.9) to be 30 kcal/kg/day and 25 kcal/kg/day for overweight or obese adolescents (P-BMI ≥ 25).^4^

Insulin was started with a dose of 0.3 IU/kg using soluble and lente forms in multiple daily injections regimen with 10-15% dose titration every 1-2 weeks to maintain the above-mentioned target glucose readings. The insulin dose adjustment decision was made by the attending obstetrician during regular antenatal care (ANC) visits (every two weeks till 36 week`s gestation and once weekly later on until delivery) or mobile phone calls made by the researchers in between the visits. Hypoglycemic episodes were reported several times during study period in the insulin group of involved women.

MTF dose was one tablet containing 500 mg given after meals three times daily. The maximum dose was 2000 mg/ 24 hours as needed. Also, using computer randomization, adolescent pregnant mothers without gestational diabetes were included as control group. All included women were followed-up until delivery through regular ANC visits (every two weeks till 36 week`s gestation and once weekly later on until delivery) or mobile phone calls made by the researchers in between the visits. MTF was well tolerated by most of the involved women in the MTF group, and only 2 women had poor MTF tolerance because of severe nausea and vomiting. These two women were switched to insulin therapy and dropped out from the study calculations.

Full medical history and examination were done during these visits and routine basic investigations were performed such as blood sugar, hemoglobin levels (Hb), and general urine analysis. Neonatal events were observed and managed by the attending neonatologist who fully examined the newborns. Compliance to lifestyle, insulin, and MTF was insured by the attending obstetrician throughout the above-mentioned ANC visits in addition to the mobile phone calls made by the researchers in between the visits.

**Fig.1:**
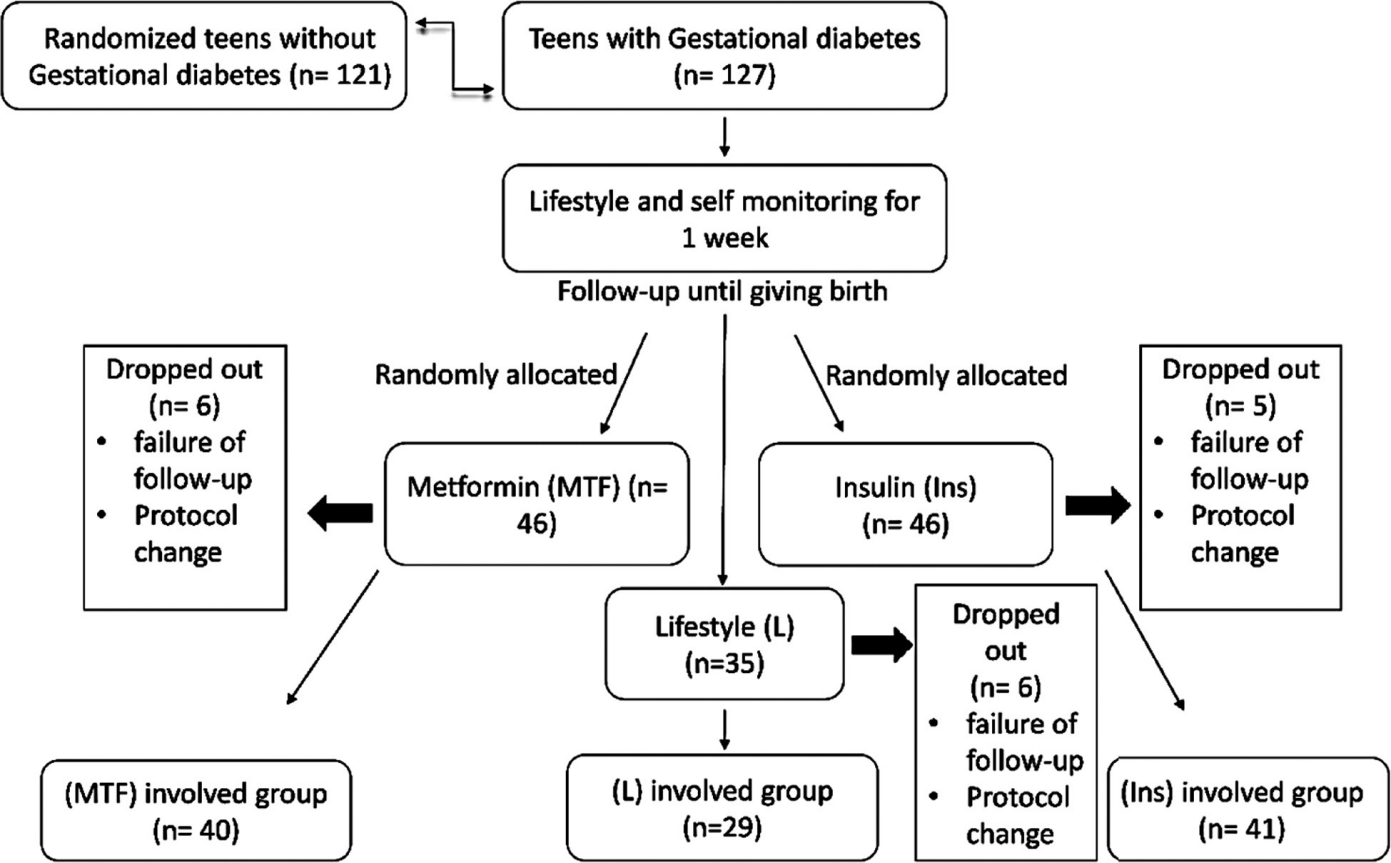
Scheme of participants.

Failure of follow-up or switching of treatment protocols during study period were considered as exclusion criteria. Flowchart in Figure 1 shows that clearly.

### Important definitions:^4^-^6^

### BMI

body weight (kg)/ height in square meters (m^2^).

### Preeclampsia

occurred when blood pressure more than 140/90 mm mercury (Hg) and proteinuria more than 0.3 gram per day.

### Large for gestational age (LGA)

birth weight > 90^th^ percentile of the mean.

### Small for gestational age (SGA)

birth weight < 10^th^ percentile of the mean.

### Neonatal hypoglycemia

venous plasma glucose < 45 mg/ dl after delivery.

### Statistical Analysis

Statistical Package for the Social Sciences version 22 was utilized for statistical analysis. Categorical parameters were expressed as a percentage, while continuous samples were expressed as mean ± standard deviation (SD). Repeated-measures ANOVA test was used to complete the analysis and comparison. Multiple logistic regression models with multivariable analysis were applied to obstetrical and neonatal events. The obstetrical model was adjusted for possible confounding factors including maternal age, P-BMI gestational age at involvement, gestational weight gain, family history of diabetes mellitus and hypertension, education level, consanguinity, residence, and presence of polycystic ovary syndrome. The neonatal model was adjusted for maternal age, P-BMI, gestational age at involvement, gestational weight gain, family history of diabetes mellitus and hypertension, education level, consanguinity, residence, polycystic ovary syndrome, preeclampsia, cesarean section (CS), and neonatal weight at birth. Significant levels were considered when p value < 0.05.

### Ethical Statement

Scientific and ethical committees located at College of Medicine, and Al-Kindy College of Medicine at University of Baghdad granted the ethical and scientific approvals (No. 1218, and 429, respectively). Informed consent was obtained from all participants in this work which was performed in line with Declaration of Helsinki.

## RESULTS

Total number of recruited adolescent pregnant women having the diagnosis of GDM was 110, divided into three main groups according to treatment plan (lifestyle= 29, MTF= 40, and insulin= 41). Another 121 adolescent pregnant women without GDM were involved as control group.

For the three major groups of GDM (lifestyle, MTF, and insulin), all the general characteristics were matched and comparable (p ≥ 0.05) as seen in Table-I. This is also applied to both GDM and control women who had matched (p ≥ 0.05) general characteristics at baseline of recruitment and afterwards except for family history of diabetes that was significantly (p < 0.05) found in GDM women than non-GDM women.

**Table-I:** General Overview of Maternal Characteristics at Recruitment for GDM Groups.

Characteristics	MTF ^a^, n= 40	Insulin, n= 41	Lifestyle, n= 29	MTF ^a^/ Insulin: p value	MTF ^a^/ Lifestyle: p value	Insulin/ Lifestyle: p value
Maternal age (years), mean ± SD ^b^	16.3 ± 2.4	16.4 ± 2.8	16.4 ± 1.1	0.78	0.34	0.82
Pre-pregnancy Body mass index (BMI), mean ± SD ^b^	28.9 ± 4.8	28.6 ± 5.3	28.5 ± 4.6	0.46	0.27	0.75
Gestational weight gain (kg), mean ± SD ^b^	8.8 ± 6.2	8.9 ± 5.6	9.1 ± 4.3	0.88	0.32	0.59
Diabetes mellitus family history, n (%)	20 (50.00%)	21 (51.22%)	16 (55.17%)	0.69	0.24	0.30
Hypertension family history, n (%)	17 (42.50%)	18 (43.90%)	13 (44.82%)	0.37	0.52	0.49
***Education level: n (%)***						
Literate	30 (75.00%)	29 (70.73%)	21 (72.41%)	0.28	0.31	0.26
Illiterate	10 (25.00%)	12 (29.27%)	8 (27.59%)			
Consanguinity, n (%)	15 (37.50%)	17 (41.46%)	12 (41.38%)	0.19	0.34	0.93
***Residence: n (%)***						
Rural	27 (67.50%)	26 (63.41%)	18 (62.07%)	0.26	0.23	0.68
Urban	13 (32.50%)	15 (36.59%)	11 (37.93%)			
Gestational age (weeks) at involvement, mean ± SD ^b^	29.8 ± 5.3	29.3 ± 4.8	28.9 ± 5.6	0.16	0.11	0.18
Presence of polycystic ovary syndrome, n (%)	4 (10.00%)	4 (9.76%)	3 (10.34%)	0.94	0.81	0.90

a: Metformin; b: Standard deviation.

In Table-II, odds ratio (OR) and 95% confidence interval (CI) were calculated. MTF had protective association (OR < 1) without being significant (p ≥ 0.05) in fasting blood sugar (FBS), random blood sugar (RBS), and gestational age (GA) at delivery in comparison to lifestyle, while MTF had significant protective association (OR= 0.76, and p < 0.05) with preeclampsia only. More details are seen in Table-II.

**Table-II:** Pregnancy Events of Each Treatment Group.

Variables	Odds ratio (95% CI) ^a^	P value
***Fasting blood sugar:***		
Lifestyle	Ref ^b^	Ref^ b^
MTF ^c^	0.95 (0.33 - 1.83)	0.35
Insulin	1.41 (1.06 - 2.74)	< 0.05
***Random blood sugar:***		
Lifestyle	Ref ^b^	Ref ^b^
MTF ^c^	0.84 (0.43 - 1.56)	0.29
Insulin	1.57 (1.21 - 3.14)	< 0.05
***Gestational age at delivery:***		
Lifestyle	Ref ^b^	Ref ^b^
MTF ^c^	0.89 (0.28 – 1.46)	0.74
Insulin	0.56 (0.11 – 1.25)	0.16
***Preeclampsia:***		
Lifestyle	Ref ^b^	Ref ^b^
MTF ^c^	0.76 (0.38 – 0.93)	< 0.05
Insulin	1.23 (0.64 - 3.82)	0.48
***Cesarean section:***		
Lifestyle	Ref ^b^	Ref ^b^
MTF ^c^	1.16 (0.52 – 1.71)	0.82
Insulin	0.94 (0.49 – 1.86)	0.51

a: Adjusted obstetrical model; b: Reference value; c: Metformin.

Moreover, gestational age (GA) estimations at delivery (weeks) presented as mean ± SD were comparable for lifestyle, MTF, and insulin women (38.7 ± 1.6, 38.6 ± 1.4, and 37.2 ± 1.8, respectively) showing slightly lower values in insulin group. CS rates for the above-mentioned treatment groups were [n= 16 (55.17%), n= 21 (52.50%), and n= 25 (60.98%), respectively]. The above-mentioned results do not appear in the tables. Neonatal complications in treatment groups are discussed in Table-III such as preterm birth, SGA, hypoglycemia, 5 minutes Apgar score > 7, and admission to Neonatal Intensive Care Unit (NICU).

**Table-III:** Neonatal Events of the Treatment Groups.

Variables	Odds ratio (95% CI) ^a^	P value
***Preterm birth:***		
Lifestyle	Ref ^b^	Ref ^b^
MTF ^c^	0.83 (0.39 - 1.45)	0.24
Insulin	1.89 (1.45 - 3.74)	< 0.05
***Large for gestational age:***		
Lifestyle	Ref ^b^	Ref ^b^
MTF ^c^	0.45 (0.20 – 0.91)	< 0.05
Insulin	1.36 (0.68 – 2.82)	0.56
***Small for gestational age:***		
Lifestyle	Ref ^b^	Ref ^b^
MTF ^c^	1.28 (0.69 – 3.35)	0.61
Insulin	2.53 (1.57 – 4.87)	< 0.05
***Hypoglycemia:***		
Lifestyle	Ref ^b^	Ref ^b^
MTF ^c^	1.37 (0.29 – 2.86)	0.59
Insulin	2.84 (1.75 – 5.22)	< 0.05
***5 minutes Apgar score > 7:***		
Lifestyle	Ref ^b^	Ref ^b^
MTF ^c^	0.94 (0.56 – 1.81)	0.73
Insulin	0.68 (0.36 – 0.92)	< 0.05
***Admission to Neonatal Intensive Care Unit:***		
Lifestyle	Ref ^b^	Ref ^b^
MTF ^c^	0.34 (0.17 – 0.89)	< 0.05
Insulin	1.26 (0.75 – 3.12)	0.13

a: Adjusted neonatal model; b: Reference value; c: Metformin.

## DISCUSSION

High-quality evidences are insufficient to determine the differences among various GDM treatment plans to have a clear decision during clinical practice.^7^ However, our search did not find such a comparison in teenage GDM.

Although not significant, P-BMI was higher and gestational weight gain was lower in our adolescent GDM pregnant women than non-GDM adolescent pregnant controls, and this was also found in MTF group when compared to lifestyle and insulin groups which may be considered as a privilege. Pregnancy by itself is a state of body insulin resistance that is increased with increasing weight. MTF has a positive influence on insulin sensitivity which in turn may improve insulin resistance leading to a better control over blood glucose during pregnancy. This idea was endorsed by many reporters.^8^

In GDM, it is preferred to slow down the curve of gestational weight gain as more kilograms during pregnancy would deepen insulin resistance affecting sugar control in a bad way.^9^ Herein this study, data revealed superior serum glucose control in MTF and lifestyle adolescent women than insulin group with the best overall fasting and postprandial serum glucose readings were found in MTF adolescents that might mirror the best improvement of insulin resistance which is one of the major factors causing GDM as indicated by other scientists who noted that MTF had faster and better sugar control while pregnant ladies might need time to be accustomed to the insulin dosage and timing.^10^

Preeclampsia in MTF group had significantly the lowest occurrence than other treatment groups. This was considered by some workers who claimed MTF as a protective agent. 11 However, a previously published paper did not agree with this finding.^12^ Many factors could affect adolescent preeclampsia that may explain these differences such as maternal age, weight, and endothelial abnormalities resulted from glucose vacillations during pregnancy.^13^ Regarding our sample, age and P-BMI were matched for the all, while our insulin adolescents had a risk factor of preeclampsia having more fluctuations in glycemic readings than other groups as noticed during the study period.

High rates of operative delivery by CS were found in all treatment groups without significant differences. These rates were higher than in other studies^14^,^15^ but closer to local CS rates regardless of age and GDM.^16^

MTF group had a significant lower rate of LGA newborns. A Finish report supported our results 17 but counteracted by another study^14^ in which the involved MTF group was not pure because of supplemented insulin doses.

### Limitations of the study

It included the small number of involved adolescent pregnant women with no full randomization. However, most of basic general features of our enrolled cases were matched at time of recruitment and it is well-known that the prevalence rate of teenage pregnant women with GDM is very low (1.33%) as estimated by a previous study.^17^

Unfortunately, although we had conducted this study throughout four years in a major tertiary center, the sample size was not large enough to perform full randomization. It is usual during teenage period to have a difficult-to-satisfy personality, disobey medical advice about diet and lifestyle changes, and fail to strictly follow the invasive approach of multi-injection insulin therapy.^18^ Accordingly, it sounds logical that oral MTF would be more satisfying than other options.^14^

## CONCLUSION

MTF treatment option for adolescent GDM had lower rates of maternal and neonatal complications when compared with other treatment plans including lifestyle and insulin.

### Author’s Contribution:

**SKJ:** Conceived, designed, and did statistical analysis, editing of manuscript, data collection and manuscript writing. She is responsible for the accuracy and integrity of work.

**HAM:** Did data collection, manuscript writing, and manuscript review.

**MAW:** Did data collection, and manuscript writing.
